# Anatomical and physiological responses of roots and rhizomes in *Oryza longistaminata* to soil water gradients

**DOI:** 10.1093/aob/mcae131

**Published:** 2024-08-10

**Authors:** Zhiwei Song, Chen Lin, Ole Pedersen, Juan Jiménez

**Affiliations:** The Freshwater Biological Laboratory, Department of Biology, University of Copenhagen, Universitetsparken 4, 3rd floor, 2100 Copenhagen, Denmark; Key Laboratory of Plant Functional Genomics of the Ministry of Education, Yangzhou University, Yangzhou 225009, China; The Freshwater Biological Laboratory, Department of Biology, University of Copenhagen, Universitetsparken 4, 3rd floor, 2100 Copenhagen, Denmark; School of Biological Sciences, The University of Western Australia, 35 Stirling Highway, Crawley, WA 6009, Australia; The Freshwater Biological Laboratory, Department of Biology, University of Copenhagen, Universitetsparken 4, 3rd floor, 2100 Copenhagen, Denmark

**Keywords:** Apparent permeance to O_2_, cortex to stele ratio, drought, flooding, large gas spaces, number of vascular bundles, perennial rice, physiological integration, radial water loss, red rice, root porosity, tissue diameter

## Abstract

**Background and Aims:**

Roots and rhizomes are crucial for the adaptation of clonal plants to soil water gradients. *Oryza longistaminata*, a rhizomatous wild rice, is of particular interest for perennial rice breeding owing to its resilience in abiotic stress conditions. Although root responses to soil flooding are well studied, rhizome responses to water gradients remain underexplored. We hypothesize that physiological integration of *Oryza longistaminata* mitigates heterogeneous water-deficit stress through interconnected rhizomes, and both roots and rhizomes respond to contrasting water conditions.

**Methods:**

We investigated the physiological integration between mother plants and ramets, measuring key photosynthetic parameters (photosynthetic and transpiration rates and stomatal conductance) using an infrared gas analyser. Moreover, root and rhizome responses to three water regimes (flooding, well watered and water deficit) were examined by measuring radial water loss and apparent permeance to O_2_, along with histochemical and anatomical characterization.

**Key Results:**

Our experiment highlights the role of physiological integration via interconnected rhizomes in mitigating water-deficit stress. Severing rhizome connections from mother plants or ramets exposed to water-deficit conditions led to significant decreases in key photosynthetic parameters, underscoring the importance of rhizome connections in bidirectional stress mitigation. Additionally, *O. longistaminata* rhizomes exhibited constitutive suberized and lignified apoplastic barriers, and such barriers were induced in roots in water stress. Anatomically, both rhizomes and roots respond in a similar manner to water gradients, showing smaller diameters in water-deficit conditions and larger diameters in flooding conditions.

**Conclusion:**

Our findings indicate that physiological integration through interconnected rhizomes helps to alleviate water-deficit stress when either the mother plant or the ramet is experiencing water deficit, while the counterpart is in control conditions. Moreover, *O. longistaminata* can adapt to various soil water regimes by regulating anatomical and physiological traits of roots and rhizomes.

## INTRODUCTION

The water regime largely determines productivity and adoption of rice, where lowland or upland rice varieties are selected and cultivated based on their outstanding performance in specific conditions of flooding or drained soils, respectively. Perennial rice, developed by hybridization of annual domesticated Asian rice (*Oryza sativa*) and its rhizomatous perennial African relative, *Oryza longistaminata*, has recently appeared as a sustainable yet productive rice alternative with great potential for cultivation in extensive areas, allowing minimum intervention without the need to replant the crop every season (Zhang *et al.*, 2023). Although some efforts have been made to improve flooding or water-deficit tolerance of perennial rice through breeding (Hu *et al.*, 2003; Soto-Gómez and Pérez-Rodríguez, 2022; Dossou-Yovo *et al.*, 2024), little is known about the concurrent root and rhizome mechanisms of adaptation to different water conditions.

Perennial rice develops a robust and extensive root system along with rhizomes containing axillary buds capable of developing roots, secondary rhizomes or ramets. In contrast to the well-studied anatomical and morphological responses of rice roots to flooding or water deficit, information regarding the rhizome responses to such conditions is limited. In roots, the formation of aerenchyma (large gas-filled spaces) increases in response to flooding or water deficit, facilitating O_2_ diffusion in the former (Yamauchi and Nakazono, 2022) or reducing the metabolic costs for resource exploration in the latter (Zhu *et al.*, 2010; Lynch *et al.*, 2014). Moreover, aerenchyma formation has been shown to increase radial water loss (RWL) in roots of rice in dry conditions (Song *et al.*, 2023). In flooded soils, the root diameter is often thicker, allowing more spaces to form aerenchyma (Yamauchi *et al*., 2013), whereas roots can be thinner in water-deficit conditions (Azhiri-Sigari *et al.*, 2000), thus increasing the capacity to explore for water. With thinner root diameters, longer roots can be formed with the same investment in resources, and also increase the surface area in contact with the soil (Comas *et al.*, 2013).

Rhizomes of *O. longistaminata* are characterized by several nodes and internodes, with a dense cortex structure, aerenchyma spaces, a pith cavity and vascular bundles (He *et al.*, 2014; Bessho-Uehara *et al.*, 2018). Well-developed aerenchyma spaces are also common in rhizomes from other wetland plants, including *Phragmites australis* (Armstrong *et al.*, 1999), *Glyceria maxima* (Braendle and Crawford, 1987), *Typha domingensis* (Duarte *et al.*, 2021) and *Paspalum wrightii* (Fabbri *et al.*, 2005). Similar to roots, the aerenchyma spaces in rhizomes can also increase in response to flooding conditions, as shown for *Sporobolus virginicus* (Naidoo and Naidoo, 1992), *Carex rostrata* (Fagerstedt, 1992), *Paspalum wrightii* (Fabbri *et al.*, 2005) and *Phragmites australis* (Danin and Naenny, 2008). However, information about the rhizome plasticity of *O. longistaminata* in response to varying water stress conditions is lacking.

Outer apoplastic barriers, composed of deposits of suberin and lignin in root cell walls of the exodermis or sclerenchyma, are commonly formed in response to flooding or water-deficit conditions (Peralta Ogorek *et al.*, 2024*a*). These outer apoplastic barriers restrict radial O_2_ loss (ROL) from roots to the rhizosphere, enhancing longitudinal O_2_ diffusion towards the root tip of plants grown in flooded soils (Jiménez *et al.*, 2021*b*). Interestingly, these outer apoplastic barriers also decrease RWL in rice roots (Song *et al.*, 2023; Peralta Ogorek *et al.*, 2024*b*). A barrier to impede ROL has been characterized in roots of *O. longistaminata* when grown in stagnant conditions (Tong *et al.*, 2023). Although information about such barriers in rhizomes is not available, the lignification of rhizomes of *Oryza coarctata* (Rajakani *et al.*, 2022) suggests that the permeability to O_2_ or water in these tissues can be limited.

Rhizomes not only have roles in storing energy and facilitating vegetative reproduction, but also serve to exchange water, nutrients and signalling molecules between mother plants and ramets (Guo *et al.*, 2021). Therefore, rhizomes can play an important role in plant adaptation to abiotic stress conditions. The clonal integration between a stressed mother plant and a ramet with sufficient resources (or vice versa), via the rhizome, can efficiently mitigate heterogeneous water stress or nutrient deficit (Kroon *et al.*, 1996; Touchette *et al.*, 2012; Tian *et al.*, 2023). In the rhizomatous invasive grass *Imperata cylindrica*, the translocation of photosynthates between mother plants and ramets enhances its invasive behaviour (Estrada *et al.*, 2020). Moreover, nitrogen translocation in the clonal plant *Carex flacca* between mother plants and ramets is similar to the pattern of water transport (Kroon *et al.*, 1998). Furthermore, clonal integration in *Carpobrotus edulis* alleviates drought stress-induced damage to photochemical activity in ramets via interconnected rhizomes and, thereby enhancing their growth (Lechuga-Lago *et al.*, 2016).

Understanding the concurrent plasticity of both root and rhizome traits of perennial rice in response to different water conditions (i.e. flooding, well watered and water deficit) will serve as a basis for developing cultivars adapted to such conditions. We hypothesized that clonal integration helps to alleviate water-deficit stress within the clone and that both roots and rhizomes can respond to soil water gradients. We therefore studied the physiological integration via interconnected rhizomes when only the mother plant or the ramet was grown in water-deficit conditions. We measured the photosynthetic parameters (photosynthetic and transpiration rates and stomatal conductance) of *O. longistaminata* before and after severing the interconnected rhizomes when mother plants or ramets were exposed to water-deficit stress. We found that the physiological integration of *O. longistaminata* via interconnected rhizomes mitigates the water-deficit stress when the mother plant or the ramet is in water-deficit conditions. We grew *O. longistaminata* in flooding, well-watered or water-deficit conditions and measured RWL and the apparent permeance to O_2_. We conducted histochemical staining and used an apoplastic tracer to visualize outer apoplastic barriers, and we characterized the anatomy of roots and rhizomes in varying water stress conditions. We found that rhizomes have constitutive barriers impeding water movement and O_2_ diffusion and that these barriers are inducible in roots when grown in flooding, well-watered or water-deficit conditions.

## MATERIALS AND METHODS

### Physiological integration via the interconnected rhizomes

In order to investigate the physiological integration via interconnected rhizomes in perennial wild rice, we designed an experiment to measure photosynthetic rate, transpiration rate and stomatal conductance before and after cutting the sympodial rhizomes when the mother plant or the ramet had been exposed to severe water deficit and its counterpart was in control conditions. Six rhizomes were collected from the growth container and were propagated in non-aerated full-strength nutrient solutions (for details of the nutrient solutions, see section below on root and rhizome responses to water gradients). Furthermore, plants composed of one mother plant and one ramet, interconnected via rhizomes, were grown separately in two independent 3-L pots. All roots, developed from either the mother plant or the ramet, were contained within their respective pots. After the ramet had developed three leaves (~8 weeks after collection of rhizomes), either the ramet or the mother plant was exposed to severe water deficit by supplementing the non-aerated nutrient solution with 20 % (w/v) PEG6000. Meanwhile, the respective counterpart ramet or mother plant continued to grow in a full-strength non-aerated nutrient solution without PEG6000. Treatments lasted for 2 weeks, and each treatment had three replicates. Photosynthetic and transpiration rates and stomatal conductance were measured in the first fully expanded leaf of either the mother or the ramet for each combination of plants exposed–unexposed to PEG6000, using an infrared gas analyser system (LI-6800, LI-COR Biosciences Inc., Lincoln, NE, USA). Measurements were collected at a photosynthetically active radiation of 800 µmol photons m^−2^ s^−1^, leaf temperature of 28 °C, CO_2_ concentration of 450 ppm and relative humidity of 70–80 %. The measurements were taken between 11:00 and 14:00 h. Measurements were collected in both mother and ramet plants 10 min before the connecting tissue was severed and 1.5 h after cutting, in 30 min intervals.

### Root and rhizome responses to water gradients

Fifteen rhizomes of *O. longistaminata* were collected from 12-month-old plants grown in an 80-L container filled with potting mix soil. Rhizomes were washed with deionized water and transplanted into pots filled with full-strength aerated nutrient solution for 2 weeks to allow propagation. The nutrient solution composition (mm) was 1.5 CaSO_4_·2H_2_O, 7.5 MES (buffer), 0.4 MgSO_4_·7H_2_O, 3.75 KNO_3_, 0.625 NH_4_NO_3_, 0.1 Na_2_O_3_Si·5H_2_O, 0.05 Fe-EDTA and 1.0 micronutrients. Micronutrients (mm) were as follows: 0.05 KCl, 0.025 H_3_BO_3_, 0.002 MnSO_4_·H_2_O, 0.002 ZnSO_4_·7H_2_O, 0.0005 CuSO_4_·5H_2_O, 0.0005 Na_2_MoO_4_·2H_2_O and 0.001 NiSO_4_·7H_2_O (pH adjusted to 6.5 using 2 m KOH). Two weeks after propagation, the 15 seedlings that emerged from rhizomes were carefully transplanted into 0.8-L rhizoboxes filled with sand culture (Supplementary Data Fig. S1). The rhizoboxes were irrigated using a dripping system containing 20 % strength nutrient solution, and the seedlings were maintained in these conditions for an extra 1 month of establishment.

After 1 month of establishment (1.5 months after rhizome collection), plants were randomly selected, and three water treatments were conducted: flooding, well watered and water deficit. For the flooding treatment, rhizoboxes were inserted into a plastic tank and filled with a 0.1 % (w/v) starch solution until reaching a water table of 3 cm above the sand surface. The application of starch solution accelerates microbial growth and thereby the consumption of O_2_ in the porewater (Mano and Omori, 2013). For the well-watered treatment, rhizoboxes were irrigated using the dripping system, whereas for the water-deficit treatment, the dripping system was removed, the rhizoboxes were put into a big plate with DI water, and the water depth was 3 cm from the bottom. Owing to the capillary force among sand granules in the water-deficit treatment, the plants can keep relatively low water availability. Rhizoboxes were organized in a completely randomized design with three treatments (flooding, well-watered and water deficit) and five replications. Treatments lasted for 3 weeks, during which the plants were irrigated daily with 20 % strength nutrient solution for the well-watered treatment. For the flooding treatment, 20 % strength nutrient solution was added weekly to the plastic tank to compensate for the water and nutrients consumed by the plants. For the water-deficit treatment, 20 % strength nutrient solution was added weekly to the big plate to compensate for the water and nutrients consumed by the plants.

### Anatomical analyses

We evaluated the anatomical changes of roots and rhizomes of plants grown in the three different water treatments. Cross-sections from roots and from the first, second and third rhizome internodes were prepared for anatomical analyses. Roots 100–110 mm in length were harvested from the plants and cut at positions of 5, 20, 40, 60 and 80 mm behind the apex using a sharp razor blade. Root segments were imbedded in 5 % (w/v) agar, and cross-sections were made using a vibrating microtome (Leica VT1200S, Leica Biosystems). Root cross-sections were stained with 1.5 % (w/v) Toluidine Blue O to increase contrast and were visualized under a bright field microscope (Olympus, BX60, Olympus Optional Co., Ltd, Tokyo, Japan). Total root porosity (aerenchyma and small intercellular air spaces), root diameter, number of cell files and the cortex to stele ratio (CSR) in root cross-sections were calculated by using ImageJ2 software (National Institutes of Health, Bethesda, MD, USA). Small intercellular air spaces were measured as described by Justin and Armstrong (1987) for cells with a cubic geometry. For rhizomes, cross-sections were made from 1.2, 2.4, 3.6, 4.8 and 6.0 mm behind the node at the first, second and third rhizome internodes. Rhizome diameter, large gas spaces (i.e. pith cavity and aerenchyma) and the number of vascular bundles were measured.

### Radial water loss

The RWL from roots and rhizomes of plants grown in flooding, well-watered and water-deficit conditions was measured following the protocol described by Song *et al.* (2023). For roots, fresh samples were collected from rhizoboxes and rinsed carefully with DI water to remove sand granules from the root surface. About 150–200 mg fresh mass of root segments was prepared from intact roots (100–120 mm in length) by removing lateral roots and the most apical 40 mm in the developing part of the root. The cut ends of the root segments were sealed with Vaseline and placed on a metal mesh into a 5-digits balance (Mettler Toledo Analytical Balance ME54). A filter bag with silica gel granules was located inside the balance chamber to maintain relative humidity at 18–28 %, and the measurements were conducted at a room temperature of 22–24 °C (HOBO UX100-011 Temperature and RH data logger, Onset). The loss in mass during desiccation was recorded automatically every 30 s for 1 h using the software Balancelink v.4.1.3. Changes in root diameter were recorded every 5 min by time-lapse pictures using a USB camera (Dino-Eye Eyepiece Camera) connected with the software Dino-Capture v.2.0. For rhizomes, about 150–200 mg fresh mass was collected from second internodes, the cut ends were sealed with Vaseline, the loss in mass was recorded automatically every 1 min for 3 h, and the rest of the process was as described for the roots.

Cumulative water loss (percentage of total water content) and RWL (in millimoles of H_2_O per square metre per second) were calculated based on total tissue water content and surface area, respectively. For roots, data of cumulative water loss and RWL were fitted by using a two-phase decay function, except for plants grown in flooding or water-deficit treatments, where a sixth-order polynomial curve showed the best fit. For rhizomes, a one-phase decay function was used to fit all cumulative water-loss data from the three treatments, and a sixth-order polynomial curve was used to fit the RWL data. The process of fitting data was conducted in order to identify the time at which 15 % of cumulated water was lost in roots, whereas in the rhizome we used the time at which 2 % of cumulated water was lost (Supplementary Data Fig. S2).

### Apparent permeance to O_2_

In addition to RWL, we aimed to assess quantitatively the capacity of roots and rhizomes to restrict ROL when grown in flooding, well-watered and water-deficit conditions. For this purpose, we measured the O_2_ intrusion into tissues using a O_2_ Clark-type microsensor (OX-25, Unisense A/S, Denmark) as described elsewhere (Peralta Ogorek *et al.*, 2021). Roots ~100–140 mm in length were collected, shortened to 15–20 mm segments corresponding to positions at 35–50 mm behind the root tip, and their cut ends were sealed with lanolin and fixed on a metal mesh inside an aquarium. The O_2_ microsensor was positioned midway along the segment, inserted 125–175 µm into the cortex before being submerged in an O_2_-saturated medium. For rhizomes, both apex and second internode regions were measured. Rhizome segments of 20–25 mm were collected from rhizoboxes, their cut ends were sealed with lanolin and they were fixed on a metal mesh inside an aquarium. The O_2_ microsensor was positioned midway along the segment, inserted 1200–1500 µm into the pith cavity before being submerged in O_2_-saturated medium. Two contrasting external gas pressures [partial pressure of O_2_ (*p*O_2_) 20.6 and ~60 kPa) in the medium were used for both root and rhizome O_2_ intrusion, and the O_2_ concentrations inside tissues were recorded using the software Logger (SensorTrace Suite v.3.2, Unisense A/S, Denmark).

### Histochemical analyses

Roots (100–140 mm long) and rhizomes (~100 mm long) were taken from plants grown in the three different water treatments. Cross-sections at 40 mm behind the root tip and from the second rhizome internode were obtained using a vibrating microtome (Leica VT1200S, Leica Biosystems). Lignification of cells was evident as red coloration of cross-sections stained with 5 % (w/v) phloroglucinol and 20 % (w/v) HCl as described by Vallet *et al.* (1996). Cell suberization was evident as green–yellowish fluorescence in cross-sections stained with a 0.01 % (w/v) Fluorol Yellow 088 solution dissolved in polyethylene glycol 400 for 1 h, as described elsewhere (Brundrett *et al.*, 1991). The stained cross-sections were mounted in a glass slide with 70 % (w/v) glycerol and observed in a bright field microscope (Olympus, BX60) for lignin or an epifluorescence microscope under ultraviolet light (Nikon ECLIPSE C*i*, Excitation filter Ex 365/10, Dichroic mirror DM-400, Barrier filter BA-400, camera Nikon DS-F*i*3) for suberin.

### Apoplastic tracer

The permeability of roots and rhizomes to solutes was assessed following the intrusion of periodic acid into the roots as described by Soukup *et al.* (2007). Briefly, root segments 100–110 mm in length were harvested, the apical 40 mm was removed, and the cut ends were sealed with lanolin. Root segments were incubated in 0.1 % (w/v) periodic acid for 1 h, followed by incubations in a reducing solution (1 g of potassium iodide and 1 g of sodium thiosulphate dissolved in 50 mL of DI water and acidified with 1 mL of 1 m hydrochloric acid) for 1 h at room temperature. After storing segments in DI water overnight at 4 °C, the segments were embedded in 5 % (w/v) agar for ≤3 days, and 100-μm-thick cross-sections were made using a vibrating microtome (Leica VT1200S, Leica Biosystems). Cross-sections were stained with Schiff’s reagent for 3–5 min, and periodic acid penetration was visualized as violet coloration (i.e. aldehydes generated after the lipid peroxidation caused by periodic acid) across the examined roots under a bright field microscope (Olympus, BX60). For rhizomes, segments 100–120 mm in length were harvested and the cut ends sealed with lanolin. Rhizome internode segments were incubated in 0.1 % (w/v) periodic acid for 2 h, followed by incubation in a reducing solution (see above) for 2 h at room temperature. After storing segments in DI water overnight at 4 °C, the segments were mounted vertically on the sample plate, and ~100-μm-thick cross-sections were prepared using a vibrating microtome (Leica VT1200S, Leica Biosystems). Cross-sections were stained with Schiff’s reagent for 5–10 min, and periodic acid penetration was visualized as violet coloration (see above) across the examined rhizomes under a bright field microscope (Olympus, BX60).

### Statistical analyses

GraphPad Prism software (v.9.0.0) was used for statistical analyses. For the physiological integration, differences between treatments were analysed by using repeated-measures ANOVA followed by Šídák’s test. For the anatomical analyses, RWL and the apparent permeance to O_2_, differences between treatments were evaluated by using two-way ANOVA followed by Tukey’s test. All data satisfied the assumption of normality (Shapiro–Wilk test) and homoscedasticity (Bartlett’s test) without requiring data transformation. The figure legends provide details on the tests used and the significance levels.

## RESULTS

### Physiological integration via interconnected rhizomes

**Fig. 1.**
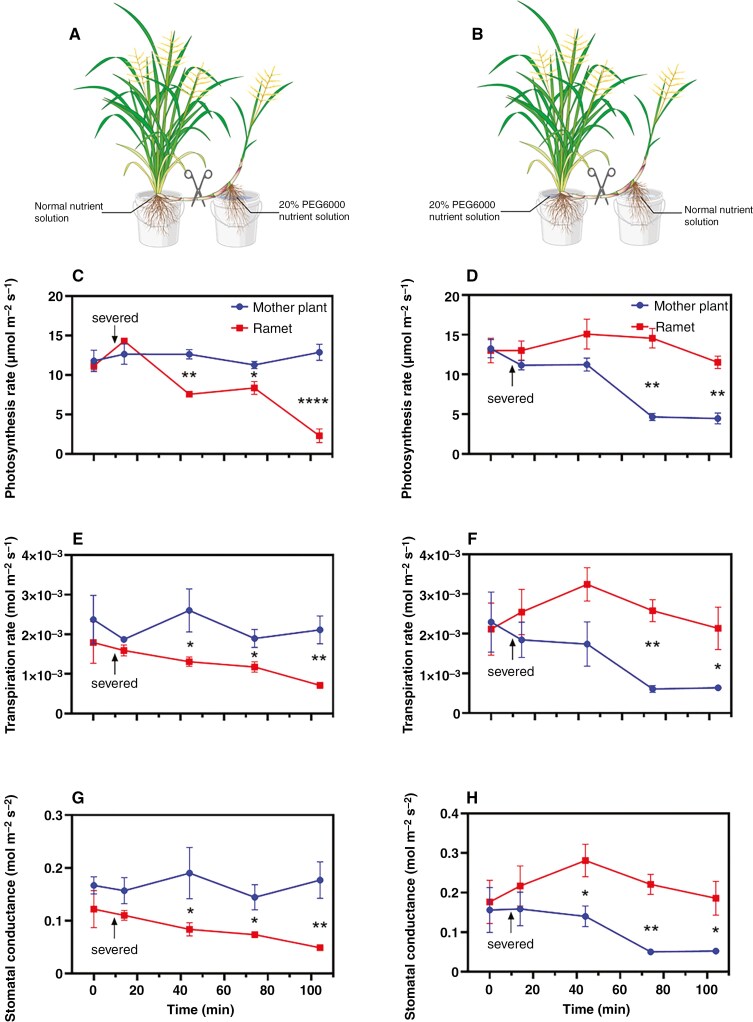
Response of key photosynthetic parameters to interconnected rhizome cutting. (A) Cutting the interconnected rhizome when the ramet is exposed to water deficit and mother plant is in control solution. (B) Cutting the interconnected rhizome when the mother plant is exposed to water deficit and the ramet is in control solution. (C, D) Photosynthetic rate. (E, F) Transpiration rate. (G, H) Stomatal conductance. Mother plant or ramet had been exposed to severe water deficit for 2 weeks, and key photosynthetic parameters were measured before and after cutting the interconnected rhizome. Black arrows indicate the time when the interconnected rhizomes were severed. Data are means ± s.e., *n* = 3. The statistical comparisons were conducted with repeated-measures ANOVA (*P* < 0.01; see Supplementary Data Table S1) followed by Šídák’s test (*P ≤ 0.05; **P ≤ 0.01; ****P ≤ 0.0001). Panels A and B created with Biorender.com.

The ability of some rhizomatous plants to alleviate stress in parts of a clone experiencing water deficit by using other parts with ample water access has been demonstrated (Kroon *et al.*, 1996; Roiloa and Retuerto, 2007; Touchette *et al.*, 2012; Lechuga-Lago *et al.*, 2016). However, for *O. longistaminata*, such physiological integration via rhizomes had not previously been established. Using key photosynthetic parameters that are particularly sensitive to water supply, we investigated the physiological integration of interconnected mother plants and ramets via rhizomes, when either of these was in water-deficit conditions and the other had ample water supply (Fig. 1A, B). We did so by cutting the sympodial rhizome while measuring, e.g. photosynthesis (*A*), transpiration (*E*) and stomatal conductance (*g*_s_). The photosynthetic and transpiration rates and the stomatal conductance of either mother plants or ramets were similar when these were interconnected via the rhizomes, irrespective of whether one of them was in stress conditions (Fig. 1). After the rhizomes connecting mother plants and ramets were cut, photosynthetic and transpiration rates and stomatal conductance decreased significantly in ramets (Fig. 1C, E, G) and mother plants (Fig. 1D, F, H) exposed to stress conditions, whereas these parameters remained stable in counterparts grown in control conditions.

These findings suggest that clonal integration is significant at the physiological level and that the integration is bidirectional, i.e. water can be supplied in both directions depending on the demand. Consequently, we expanded our approach to examine the anatomical responses of roots and rhizomes not only to water deficit but also to excess water resulting in flooding. This helped us to understand how roots and rhizomes interact in adapting the below-ground organs to gradients in soil water.

### Root and rhizome anatomical traits in response to water gradients

**Fig. 2.**
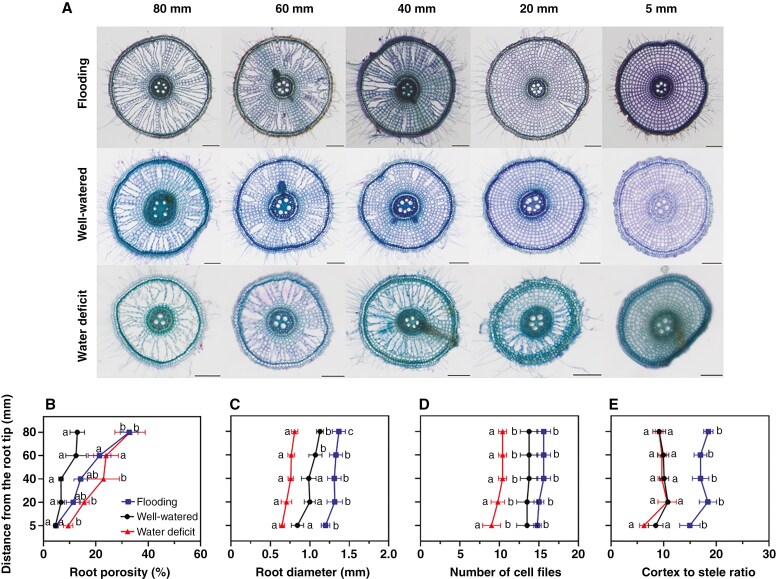
(A) Root cross-sections of *Orya longistaminata* grown in flooding, well-watered or water-deficit conditions. (B–E) Root porosity (B), root diameter (C), number of cell files (D) and cortex to stele ratio (E). Measurements were collected longitudinally at 5, 20, 40, 60 and 80 mm behind the root tip. In B, root porosity refers to both aerenchyma and small intercellular air spaces. Data are means ± s.e., *n* = 5. The statistical comparisons were conducted using two-way ANOVA (*P* < 0.01; see Supplementary Data Table S2) followed by Tukey’s test (different letters indicate significant difference, *P* < 0.05), and all data passed the Shapiro–Wilk normality test. In B, *P*_T_ = 0.0226, *P*_P_ < 0.0001 and *P*_T×P_ = 0.0489. In C, *P*_T_ < 0.0001, *P*_P_ = 0.0014 and *P*_T×P_ = 0.8465. In D, *P*_T_ = 0.0024, *P*_P_ = 0.0403 and *P*_T×P_ = 0.6660. In E, *P*_T_ < 0.0001, *P*_P_ = 0.0191 and *P*_T×P_ = 0.8690. Abbreviations: P, position; T, treatment; T × P, treatment and position interaction. Scale bars in A: 200 µm.

Anatomical traits involved in gas diffusion, water transport or resource optimization are known to respond significantly to gradients in soil water (Yamauchi *et al.*, 2021). We therefore evaluated anatomical changes at several positions behind the root tip (i.e. 5, 20, 40, 60 and 80 mm) and at the first, second and third internodes for rhizomes, in response to the three water regimes used. Root porosity (i.e. aerenchyma and small intercellular air spaces) was higher at basal regions and decreased significantly towards the root apex for all plants, independently of the water regimes (Fig. 2A, B). However, the mean root porosity from well-watered conditions (8.7 %) increased in flooding (17 %) and water-deficit conditions (21 %). Notably, roots under water deficit had 2.0-fold higher porosity at 5 mm behind the root tip than that of roots from flooding or well-watered conditions. At 80 mm behind the root tip, the root porosity responded even more, with 13 % in well-watered conditions compared with 33 % and 34 % in flooding and water-deficit conditions, respectively (Fig. 2B). The root diameter also responded to soil water content, with roots being thinner in water-deficit conditions (0.74 mm) compared with 1.00 and 1.31 mm from well-watered and flooding conditions, respectively (Fig. 2C). Likewise, roots in water-deficit conditions had a lower number of cell files, averaging 10, compared with 14 and 15 in well-watered and flooding conditions, respectively (Fig. 2D). Finally, the cortex to stele ratio (17.2) was significantly higher for roots in flooding conditions in comparison to well-watered or water deficit-conditions (9.6 vs. 9.1) (Fig. 2E).

**Fig. 3.**
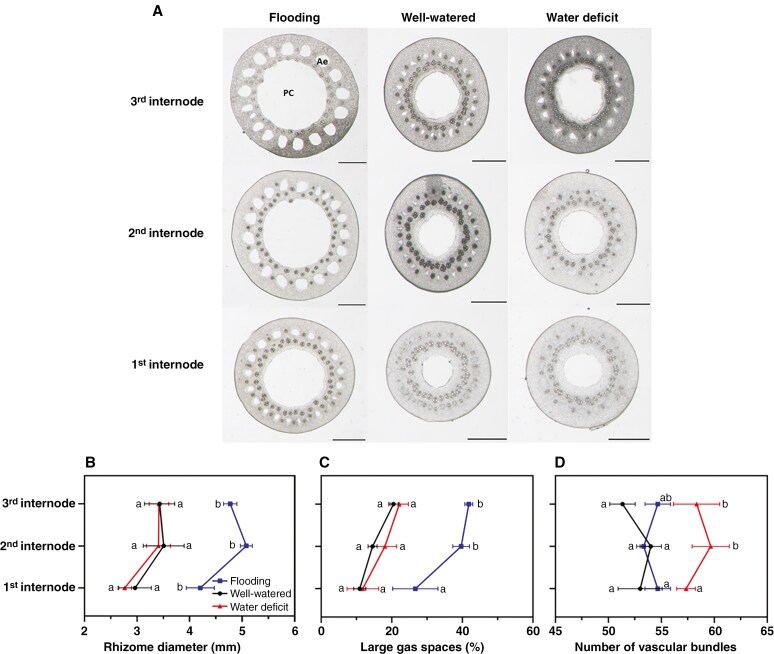
(A) Rhizome cross-sections of *Oryza longistaminata* grown in flooding, well-watered or water-deficit conditions. (B–D) Rhizome diameter (B), large gas spaces (C) and number of vascular bundles (D). Measurements were collected longitudinally at the first, second and third rhizome internodes. Refer to Supplementary Data Figure S3 for the longitudinal variation in these traits along different internodes. In C, the large gas spaces refer to both cortical aerenchyma and the pith cavity. Data are means ± s.e., *n* = 3. The statistical comparisons were conducted with two-way ANOVA (*P* < 0.01; see Supplementary Data Table S3) followed by Tukey’s test (different letters indicate significant difference, *P* < 0.05), and all data passed the Shapiro–Wilk normality test. In B, *P*_T_ = 0.0054, *P*_P_ = 0.0007 and *P*_T×P_ = 0.6208. In C, *P*_T_ = 0.0036, *P*_P_ = 0.0008 and *P*_T×P_ = 0.2165. In D, *P*_T_ = 0.0110, *P*_P_ = 0.6611 and *P*_T×P_ = 0.5312. Abbreviations: Ae, aerenchyma; P, position; PC, pith cavity; T, treatment; T × P, treatment and position interaction. Scale bars in A: 1 mm.

We found that rhizomes also responded to soil water gradients. Larger internode diameters (4.67 mm) were observed in plants grown in flooding compared with well-watered or water deficit-conditions (3.30 vs. 3.20 mm) (Fig. 3A, B). The diameter and large gas spaces (i.e. pith cavity and aerenchyma) of the first, second and third rhizome internode of plants grown in flooding were significantly higher than those from well-watered or water-deficit conditions (Supplementary Data Fig. S3). Longitudinally and in well-watered conditions, the large gas spaces increased significantly from 10.9 % at the first to 20.5 % at the third internode (Fig. 3C; Supplementary Data Fig. S3). However, the large gas spaces also increased in response to flooding (36.1 %) compared with well-watered or water-deficit conditions (15.4 vs. 17.3 %) (Fig. 3C). The mean number of vascular bundles was 54 and 53 in rhizomes from flooding and well-watered conditions, respectively, whereas it was 58 in rhizomes under soil water deficit (Fig. 3D). In contrast, the various anatomical traits along different longitudinal positions within the same rhizome internode (0–6 mm behind the node) exhibited no discernible response to different water treatments (Supplementary Data Fig. S3; Supplementary Data Table S4).

### Permeability of roots and rhizomes to water and O_2_

**Fig. 4.**
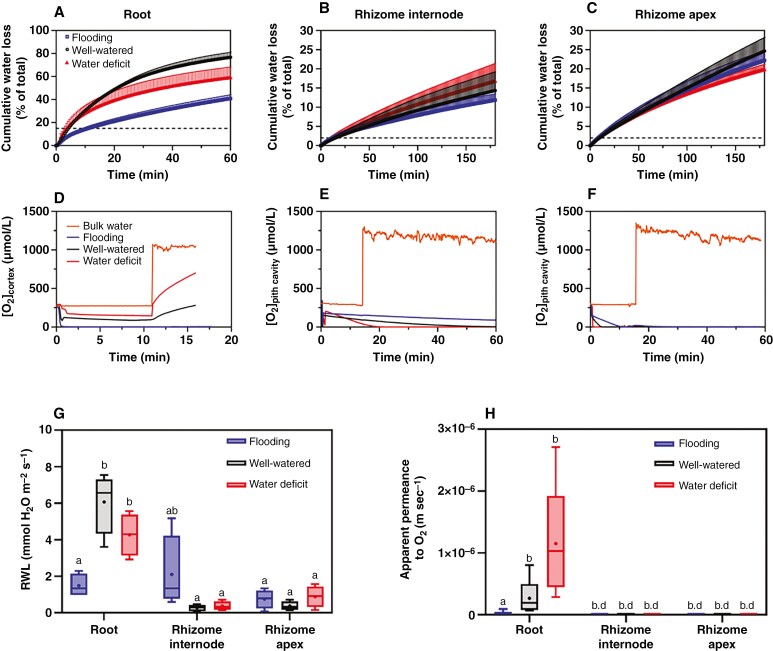
The radial water loss (RWL) and the apparent permeance to O_2_ of roots and rhizomes in *Oryza longistaminata* grown in flooding, well-watered or water-deficit conditions. (A–C) Cumulative water loss. (D–H) Time trace of O_2_ intrusion (D–F), RWL (G) and apparent permeance to O_2_ (H). The RWL values for roots were extracted at the time point at which 15 % cumulative water loss had occurred (dashed line in A and Supplementary Data Fig. S4). For rhizome internodes and apexes, RWL values were extracted at the time at which 2 % cumulative water loss had occurred (dashed line in B, C and Supplementary Data Fig. S4). For apparent permeance to O_2_, root segments of 15–20 mm, corresponding to positions at 35–50 mm behind the root tip, and rhizomes of 20–25 mm in length from the second internode or the apex were used. The statistical comparisons were conducted using two-way ANOVA (see Supplementary Data Tables S5 and S6; *P* < 0.01) followed by Tukey’s test (different letters indicate significant difference, *P* < 0.05), and all data passed the Shapiro–Wilk normality test. Data are means ± s.e., *n* = 5. Mean, +; median, horizontal line; second and third quartiles, box; minimum and maximum values, whisker. Abbreviation: b.d., below detection limit (i.e. *p*O_2_ < 0.02 kPa).

The apoplastic barrier in the outer part of the root can restrict both solute flow and gas diffusion (Ranathunge *et al.*, 2011; Peralta Ogorek *et al.*, 2021). Using RWL as a diagnostic tool, we found that adventitious roots of *O. longistaminata* formed a tight outer apoplastic barrier under flooding, whereas only a weak barrier was present in well-watered or water-deficit conditions. After 1 h of desiccation, roots growing in well-watered conditions reached 80 % of the cumulative water loss and only 40 % and 60 % in flooding and water-deficit conditions, respectively (Fig. 4A). The RWL from roots of plants grown in well-watered conditions was 4.1-fold higher compared with that of roots from flooding conditions. Likewise, RWL was 2.9-fold higher in roots from water-deficit compared with those from flooding conditions (Fig. 4G). In comparison, we found no differences in RWL from rhizome internodes or rhizome apexes for plants grown under the three soil water regimes (Fig. 4G). Furthermore, after 3 h of desiccation, the cumulative water loss reached 10–15 % in rhizome internodes grown in the three treatments, but 20–25 % in rhizome apexes (Fig. 4B, C).

After moving roots from air equilibrium to high O_2_, the cortical O_2_ concentration increased over time in roots from both well-watered and water-deficit conditions, whereas it remained below detection limit in flooding conditions (Fig. 4D). In addition, the increase in O_2_ concentration in roots grown in water-deficit conditions was much faster than that of roots grown in well-watered conditions (Fig. 4D). The apparent permeance to O_2_ in roots from well-watered and water-deficit conditions was significantly higher than that of roots from flooding conditions (Fig. 4H). For flooding conditions, the apparent permeance to O_2_ was 1.83 × 10^−8^ m s^−1^, whereas it increased 30- to 63.0-fold for plants in well-watered or water-deficit conditions, respectively. For rhizome internodes and apexes from flooding, well-watered or water-deficit conditions, the O_2_ intrusion decreased over time after moving roots to high O_2_ (Fig. 4E) or was below the detection limit (Fig. 4F), indicating that tissue O_2_ consumption exceeded O_2_ intrusion (Fig. 4H).

### Root apoplastic barriers

**Fig. 5.**
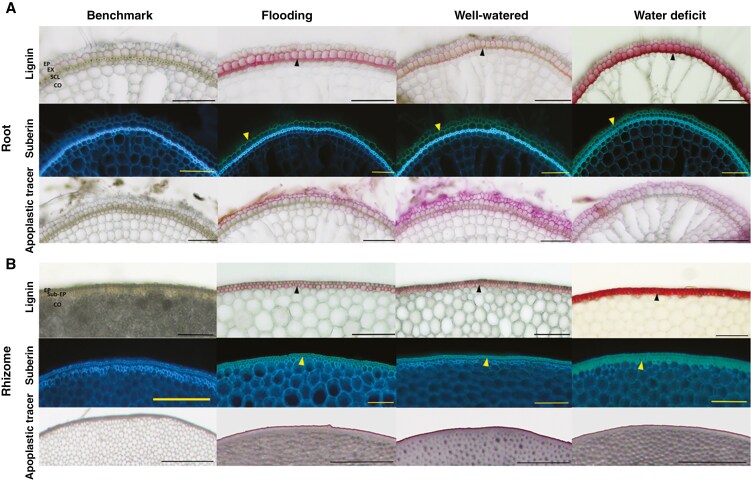
Patterns of lignification, suberization and permeability to apoplastic tracer of roots (A) and rhizomes (B) of *Oryza longistaminata* grown in flooding, well-watered or water-deficit conditions. Root cross-sections were taken at 40 mm behind the root tip and rhizome cross-sections at the second internode. Black arrowheads point at lignified exodermal and sclerenchyma cells in roots and at epidermal and sub-epidermal cells of rhizomes. Yellow arrowheads point at suberized exodermal cells in roots and at epidermal and sub-epidermal cells in rhizomes. Abbreviations: CO, cortex; EP, epidermis; EX, exodermis; SCL, sclerenchyma; Sub-EP, sub-epidermis. Scale bars in A and B: 100 µm.

Cell wall components involved in apoplastic barrier formation, such as lignin, can be visualized using histochemical staining (Liu and Kreszies, 2023). Root and rhizome cross-sections were therefore stained with phloroglucinol-HCl, which produces a red coloration in the presence of lignin (Vallet *et al.*, 1996). We found substantial lignification of sclerenchyma and exodermal cell walls regardless of soil water regime, but roots from flooding or water-deficit conditions showed stronger coloration (Fig. 5A). Suberin is also an important cell wall component in apoplastic barriers (Vishwanath *et al.*, 2015), and suberization of exodermal cells, indicated by green–yellowish fluorescence, was evident in all roots and to a similar extent regardless of water regime (Fig. 5A). Interestingly, there were several cells in the sclerenchyma and exodermis without lignin or suberin (i.e. ‘windows’), particularly in roots from well-watered or water-deficit conditions, whereas these windows were significantly fewer in roots from flooding (Supplementary Data Fig. S4). The solute permeability across the outer part of the root, investigated by penetration of periodic acid and evident as purple coloration, indicated that periodic acid penetrated the outer root cells and reached inner tissues (cortex) in roots from well-watered or water-deficit conditions, whereas the penetration stopped at the exodermis in roots from flooding (Fig. 5A).

In rhizomes, lignification was evident at epidermal and sub-epidermal cells of rhizomes from all three water regimes, but with higher coloration in rhizomes from water-deficit conditions (Fig. 5B). Moreover, suberization of epidermal and sub-epidermal cells was evident for rhizomes regardless of soil water conditions (Fig. 5B). Finally, penetration of the apoplastic tracer (periodic acid) stopped at the cortical cells of rhizomes independently of the water treatment, indicating that rhizomes of *O. longistaminata* form a constitutive apoplastic barrier in their rhizomes (Fig. 5B).

## DISCUSSION

Cultivation of perennial rice is a sustainable and productive option in many rice-growing areas, but very little is known about the mechanisms of adaptation to different water gradients of rhizome-bearing rice. This study documents significant acclimations in roots and rhizomes of the perennial rhizomatous *O. longistaminata* in response to flooding, well-watered and water-deficit conditions. The development of barriers to impede ROL and RWL is constitutive on rhizomes, whereas this trait is modulated in roots in response to water conditions. We also provide compelling evidence for the physiological integration alleviating water-deficit stress of mother or ramet plants when one is short of water. Below, we discuss these findings, with a focus on the anatomical changes in roots and rhizomes and how these traits contribute to the physiological integration under water-deficit stress.

### *The physiological integration of* O. longistaminata *mitigates heterogeneous water-deficit stress via the interconnected rhizomes*

To justify in-depth investigation of rhizome and root responses to a gradient in soil water, we initially designed an experiment to examine whether there is significant physiological integration within clones of *O. longistaminata*. We measured key photosynthetic parameters (i.e. photosynthetic and transpiration rates and stomatal conductance) in the mother plant and ramet before and after cutting the interconnected rhizomes (Fig. 1). When either the mother plant or the ramet was exposed to severe water deficit, cutting the interconnected rhizome led to significant declines in the photosynthetic parameters of the mother plant or ramet that was exposed to stress. However, the counterpart in control conditions remained unchanged (Fig. 1C–H). These results suggest an exchange of water, nutrients or molecular signals through the horizontal rhizomes. Physiological integration is one of the key traits associated with clonal growth, which allows resource sharing between interconnected ramets within a clonal system (Portela *et al.*, 2021). Through the support given by the mother plant to the ramet, clonal plants can explore new soil water resources during water deficit. Once the ramet reaches new water resources, the ramet can, in turn, support the mother plant remaining under water-deficit stress. Therefore, physiological integration can greatly enhance the ability to avoid drought in *O. longistaminata*. Notably, the ramet in the present study exhibited a swifter response to water deficit than the mother plant (Fig. 1), possibly owing to its greater reliance on the water status of the mother plant. This dependence is likely to render the ramet more sensitive to sudden water-deficit conditions than the mother plant. Our results suggest that the physiological integration of *O. longistaminata* is bidirectional via the interconnected rhizomes, making the rhizome key in mitigating heterogeneous water-deficit stress.

### Constitutive barriers developed in rhizomes, whereas this trait is modulated in roots in response to water gradients

The finding that physiological integration within a clone of *O. longistaminata* is significant merits a more thorough investigation of the underlying reasons. We found that the rhizome exhibited a constitutive barrier to ROL, with depositions of lignin and suberin observed at the epidermis and sub-epidermis in rhizomes grown in flooding, well-watered or water-deficit conditions (Figs 4H and 5B). In addition, the movements of the apoplastic tracer were blocked at the epidermis and sub-epidermis, indicating that rhizomes were radially impermeable to solutes. The constitutive barrier also significantly restricted RWL in rhizomes, as shown in flooding, well-watered or water-deficit conditions (Fig. 4G); this would have possible functional implications in a dry soil. The constitutive barrier in the outer part of the rhizome was demonstrated further by the low apparent permeance to O_2_ in all three conditions of soil water availability (Fig. 4H). In addition to the cell wall depositions, the dense cortical cells (Fig. 3A) in rhizomes could also contribute to the low apparent permeance to O_2_, because O_2_ consumption can be high and diffusivity low in bulky tissues (Jiménez *et al.*, 2024). A previous study has shown that a suberized and lignified constitutive barrier formed in adventitious roots of wild rice, *O. glumaepatula*, where it restricted ROL in aerated conditions (an experimental approach to simulate a drained soil) (Ejiri *et al.*, 2020). However, our study is the first to report the formation of constitutive barriers in rhizomes of *O. longistaminata*.

The roots of *O. longistaminata* had significantly higher RWL and apparent permeance to O_2_ in well-watered or water-deficit conditions than that in flooding conditions (Fig. 4G, H). The higher RWL and apparent permeance to O_2_ (i.e. indicating a weak barrier) could be attributable to leaks in the apoplastic barriers, influenced by more lateral roots developing and the presence of ‘windows’ of non-suberized or non-lignified cells in well-watered or water-deficit conditions (Supplementary Data Figure S4). The formation of a window would provide a radial diffusive pathway for water and O_2_. The formation of window sites has been reported in roots of several wetland plant species, such as cultivated rice (Justin and Armstrong, 1987), *Phragmites australis* (Armstrong *et al.*, 2000), *Hordeum marinum* (Garthwaite *et al.*, 2008), in which the outer apoplastic barriers can be induced, and in roots of *Urochloa humidicola* with constitutive barriers to ROL (Jiménez *et al.*, 2021*a*). In a previous study, in which roots of *O. longistaminata* induced outer apoplastic barriers when grown in stagnant, deoxygenated nutrient solutions [an experimental approach mimicking soil flooding (Wiengweera *et al.*, 1997)], the RWL was 2 mmol m^−2^ s^−1^ (Tong *et al.*, 2023), in comparison to 1.8 mmol m^−2^ s^−1^ in the present study with roots in flooded sand. Moreover, the apoplastic tracer was blocked at the outer part of roots grown in flooding conditions, whereas the tracer penetrated into the cortex in roots from well-watered or water-deficit conditions (Fig. 5A). These results show that roots of *O. longistaminata* can induce a strong outer apoplastic barrier in flooding conditions. It might be logical to assume that the formation of more windows in the outer apoplastic barrier of roots from water-deficit conditions would facilitate water uptake, in contrast to a completely sealed barrier with very few windows in flooding conditions preventing ROL to the rhizosphere.

### Key anatomical traits of roots and rhizomes respond significantly to soil water gradients

It is well known that plants can acclimate to soil water gradients via plasticity in root anatomical traits (e.g. Yamauchi *et al.*, 2021, 2024). In the present study, we found that root porosity (aerenchyma plus minor intercellular gas spaces) was higher (21.0 %) in water-deficit compared with flooding (17.0 %) or well-watered (8.7 %) conditions (Fig. 2B). This finding suggests that *O. longistaminata* could reduce energy costs for root growth through programmed cell death for adaptation to water-deficit conditions (Lynch, 2018). Moreover, thinner roots with a lower number of cell files in the cortex were formed in water-deficit conditions compared with flooding or well-watered conditions (Fig. 2C, D). This response results in a higher surface area to volume ratio, facilitating the relative contact area to the soil, and at the same time it reduces the distance for water and nutrients to move from the rhizosphere to the stele. The CSR represents the balance between cortex and stele proportions in roots, and a high CSR is beneficial for aerenchyma formation in flooding conditions (owing to more cortex tissues), whereas a low CSR facilitates water uptake in drought conditions (Yamauchi *et al.*, 2024). Here, we found that the CSR in roots from water-deficit conditions was similar to that of roots from well-watered conditions (Fig. 2E). This reduction in CSR was related to a reduced number of cell files (Fig. 2D) rather than to changes in root diameters (Fig. 2C). In contrast, the CSR of flooded roots was higher than that of roots grown in well-watered or water-deficit conditions, which might be associated with a higher number of cell files (Fig. 2D) and thicker root diameters (Fig. 2C). In summary, the roots of *O. longistaminata* exhibit significant acclimation to water-deficit conditions by increasing root porosity, decreasing root diameter and reducing the number of cell files, to lower metabolic costs and enhance water uptake. In contrast, in flooding conditions, thicker roots of high porosity facilitate oxygen diffusion from the basal to the apical regions.

Interestingly, in contrast to the constitutive nature of the outer apoplastic barriers, the anatomical features of rhizomes of *O. longistaminata* respond to soil water gradients. The rhizome internodes had significantly lower tissue diameter and lower areas of large gas spaces in water-deficit or well-watered conditions than in flooding conditions (Fig. 3B, C). This suggests that rhizomes might have lower metabolic costs in water-deficit or well-watered conditions compared with flooding conditions. Additionally, the lower area of large gas spaces could facilitate cortical carbohydrate storage for recovery after drought stress. Moreover, thicker rhizomes in flooded conditions provide more space for the formation of cortical aerenchyma (Braendle and Crawford, 1987), with the pith cavity and cortical aerenchyma in rhizomes serving as an important aeration pathway in waterlogged conditions (Armstrong *et al.*, 1992). In contrast, the increase in the number of vascular bundles in rhizomes under water deficit demonstrates that rhizomes regulate the formation of vascular bundles to enhance water transport (Fig. 3D). Finally, the constitutive barriers in rhizomes can effectively reduce water and O_2_ loss, thereby improving longitudinal fluxes to other tissues (Figs 4G, H and 5B).

Roots and rhizomes share many strategies for acclimating to water gradients, but they also differ in some aspects. Roots and rhizomes both had thinner tissues under water deficit and thicker tissue diameters in flooding conditions (Figs 2C and 3B). We propose that *O. longistaminata* increases water uptake and transport in water-deficit conditions by investing energy into tissue expansion to form a larger stele in roots (i.e. lower CSR; see Fig. 2E) and by forming more vascular bundles in the rhizomes (see Fig. 3D). However, during water deficit, the higher root porosity but much lower areas of large gas spaces in rhizomes show that roots and rhizomes have different strategies in water-deficit conditions, i.e. roots form more porosity to save resources, whereas rhizomes might require more cortical cells for carbohydrate storage for recovery after the stress (Figs 2B and 3C).

## CONCLUSION

We demonstrated that bidirectional physiological integration is significant, as indicated by the rapid changes in key photosynthetic parameters when the horizontal communication between mother plants and ramets was interrupted. We also showed that roots of *O. longistaminata* form a strong outer apoplastic barrier in flooded conditions, whereas the barrier is weak in well-watered or water-deficit conditions. The rhizomes had constitutive barriers in their outer parts, as indicated by low RWL, low apparent permeance to O_2_, histochemical staining of lignin and suberin, and limited radial movements of the apoplastic tracer. Anatomically, both roots and rhizomes were thinner during water deficit, whereas they were thicker during flooding. Compared with flooding, roots had a lower cortex to stele ratio in water-deficit conditions, and rhizomes had a higher number of vascular bundles. Interestingly, root porosity increased in flooding or water-deficit conditions, whereas large gas spaces in rhizomes decreased in well-watered or water-deficit conditions. We therefore propose that *O. longistaminata* acclimates to soil water deficits by inducing an outer apoplastic barrier in the roots to restrict radial water loss, decreasing root diameter and forming windows to improve the water uptake. The rhizomes acclimate by increasing the number of vascular bundles to enhance water transport capability and by reducing rhizome diameter to decrease growth and metabolic costs.

## SUPPLEMENTARY DATA

Supplementary data are available online at https://academic.oup.com/aob and consist of the following.

Figure S1: the growth conditions of *Oryza longistaminata* in rhizoboxes with sand culture. Figure S2: the time trace of radial water loss in roots, rhizome internode and rhizome apex of *Oryza longistaminata* grown in flooding, well-watered or water-deficit conditions. Figure S3: anatomical traits of first, second and third internodes in rhizomes of *Oryza longistaminata* grown in flooding, well-watered or water-deficit conditions. Figure S4: number of windows without lignified or suberized cells. Table S1: results of repeated-measures ANOVA of responses of photosynthetic parameters to cutting of the rhizome connecting the mother plant and the ramet in *Oryza longistaminata*. Table S2: results of two-way ANOVA of root anatomical analyses in *Oryza longistaminata*. Table S3: results of two-way ANOVA of first, second and third rhizome internodes from anatomical analyses of *Oryza longistaminata*. Table S4: results of two-way ANOVA of anatomical analyses of first, second and third rhizome internodes in *Oryza longistaminata*. Table S5: results of two-way ANOVA of radial water loss from roots and rhizomes of *Oryza longistaminata*. Table S6: results of two-way ANOVA of apparent permeance to O_2_ in roots and rhizomes of *Oryza longistaminata*.

mcae131_suppl_Supplementary_Material
