# Supplementary material for: Anatomical and physiological responses of roots and rhizomes in Oryza longistaminata to soil water gradients
Source: Ann Bot. 2024 Aug 10;136(5-6):1251–63. doi: 10.1093/aob/mcae131 (PMC12682861; doi:10.1093/aob/mcae131)
Supplement: mcae131_suppl_Supplementary_Material [file mcae131_suppl_supplementary_material.docx]

# **SUPPLEMENTARY DATA FOR Anatomical and physiological responses of roots and rhizomes in *Oryza longistaminata* to soil water gradients**

**Zhiwei Song^1,*^**, **Chen Lin^2^**, **Ole Pedersen^1,3^** and **Juan de la Cruz Jiménez^1,*^**

1) The Freshwater Biological Laboratory, Department of Biology, University of Copenhagen, Universitetsparken 4, 3^rd^ floor, 2100 Copenhagen, Denmark.

2) Key Laboratory of Plant Functional Genomics of the Ministry of Education, Yangzhou University, Yangzhou 225009, China.

3) School of Biological Sciences, The University of Western Australia, 35 Stirling Highway, Crawley, WA 6009, Australia.

**ORCID numbers**

Zhiwei Song 0000-0002-1600-1353

Chen Lin 0000-0003-3256-6415

Ole Pedersen 0000-0002-0827-946X

Juan de la Cruz Jiménez 0000-0002-9985-5302

**Corresponding authors**

Zhiwei Song: zhiwei.song@bio.ku.dk

Juan de la Cruz Jiménez: juan.jimenezserna@bio.ku.dk

Ole Pedersen: ole.pedersen@uwa.edu.au


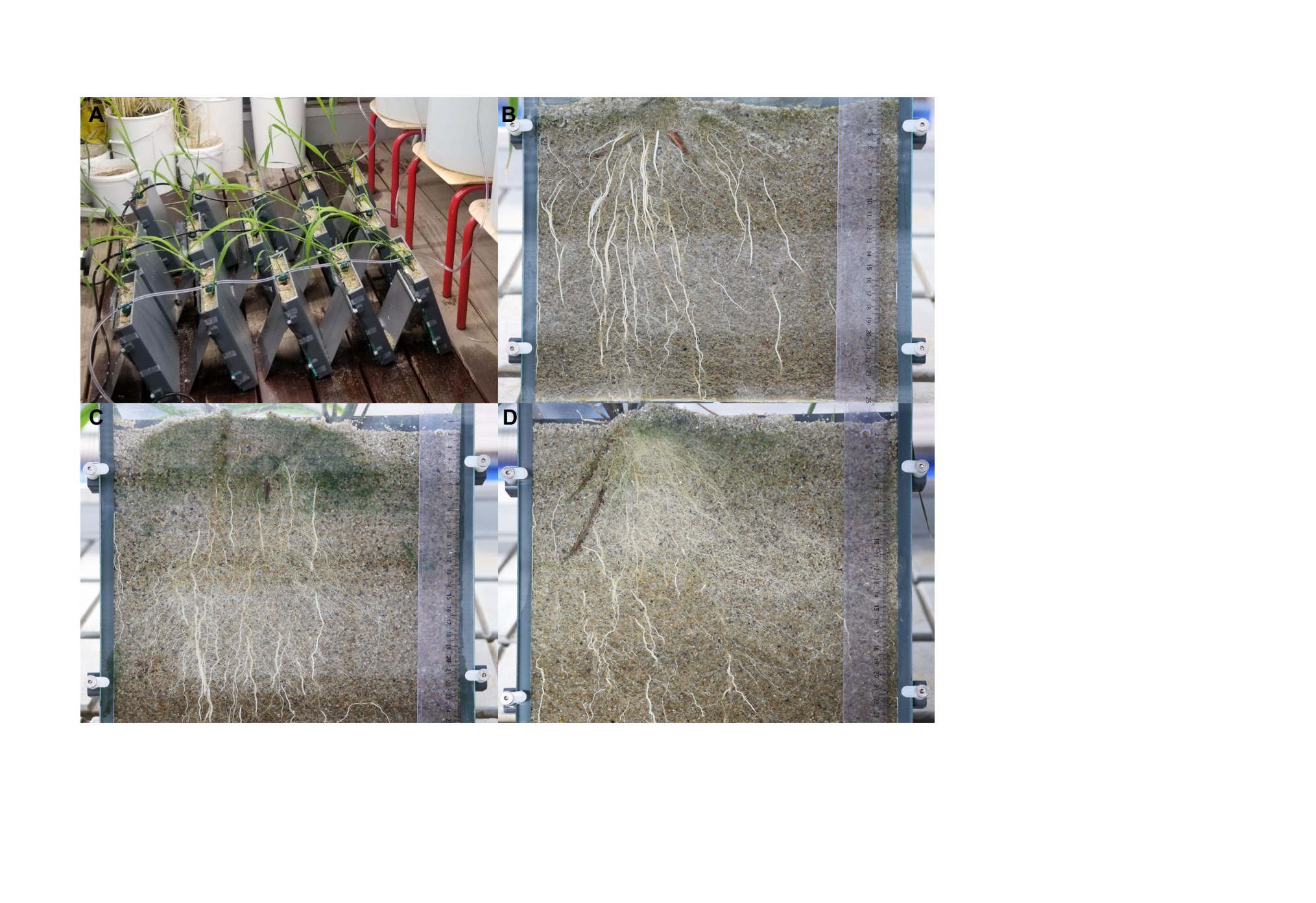


## **Figure S1**

The growth conditions of *Oryza longistaminata* in rhizoboxes with sand culture. Experimental set-up after two weeks of propagation (A). Representative images of roots from plants exposed to flooding (B), well-watered (C) and water deficit conditions (D). The rhizoboxes were irragated using a dripping system flowing a 20% strength nutrient solution. The rhizoboxes were designed as 280 mm*50 mm*290 mm (length*width*depth) in size with the removable glass lids and two drainage holes underneath the rhizoboxes.


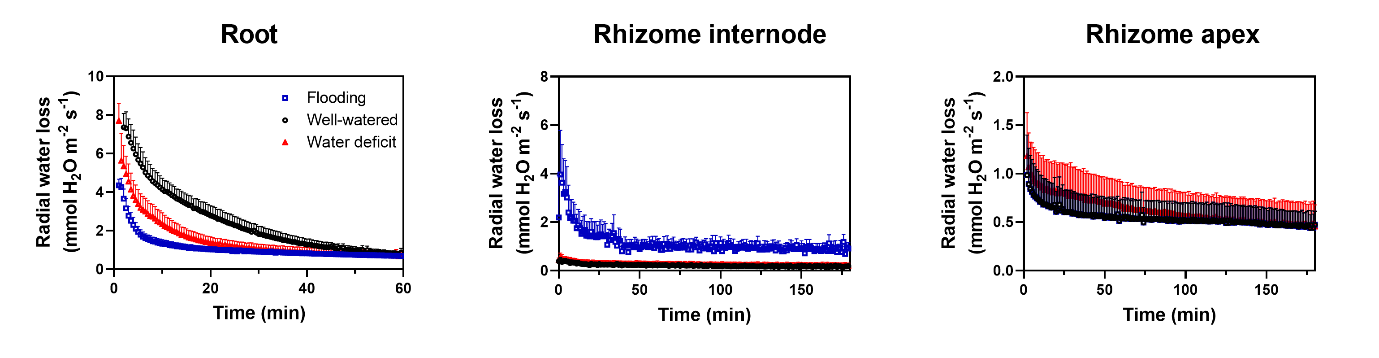


## **Figure S2**

The time trace of radial water loss in roots, rhizome internode, and rhizome apex of *O. longistaminata* grown in flooding, well-watered, or water deficit conditions. Data are means ± SE, *n*= 5.


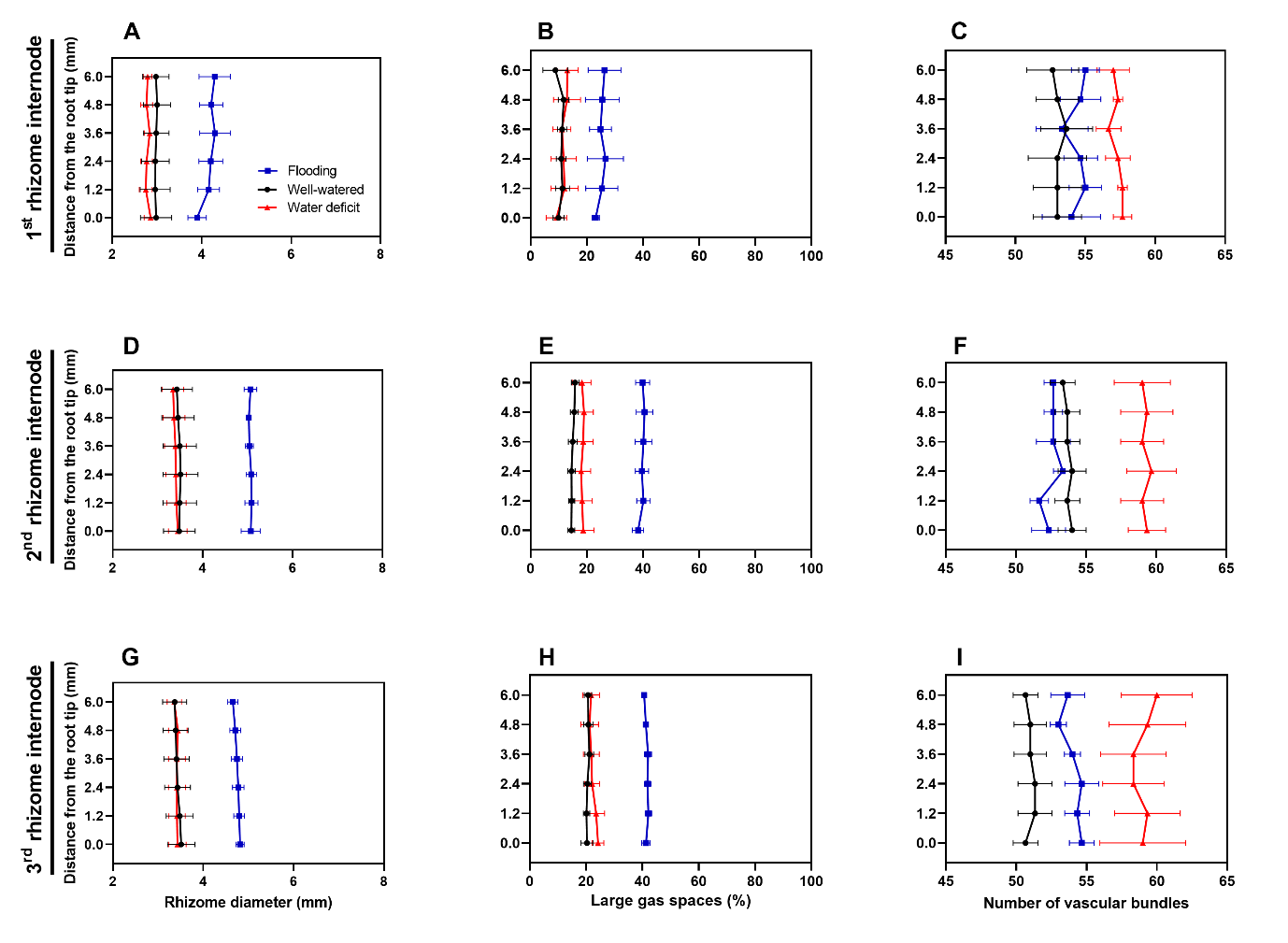


## **Figure S3**

Anatomical traits of 1^st^, 2^nd^, and 3^rd^ internodes in rhizomes of *O. longistaminata* grown in flooding, well-watered or water deficit conditions. Rhizome diameter (A, D, G), large gas spaces (B, E, H), and number of vascular bundles (C, F, I). Measurements were collected at 0, 1.2, 2.4, 3.6, 4.8, and 6.0 mm from the node. Data are means ± SE, *n*= 3. Statistical comparisons were conducted using two-way ANOVA (see supplementary data Table S4, *P* < 0.01) followed by a Tukey’s test and all data passed Shapiro-Wilk’s normality test.

##
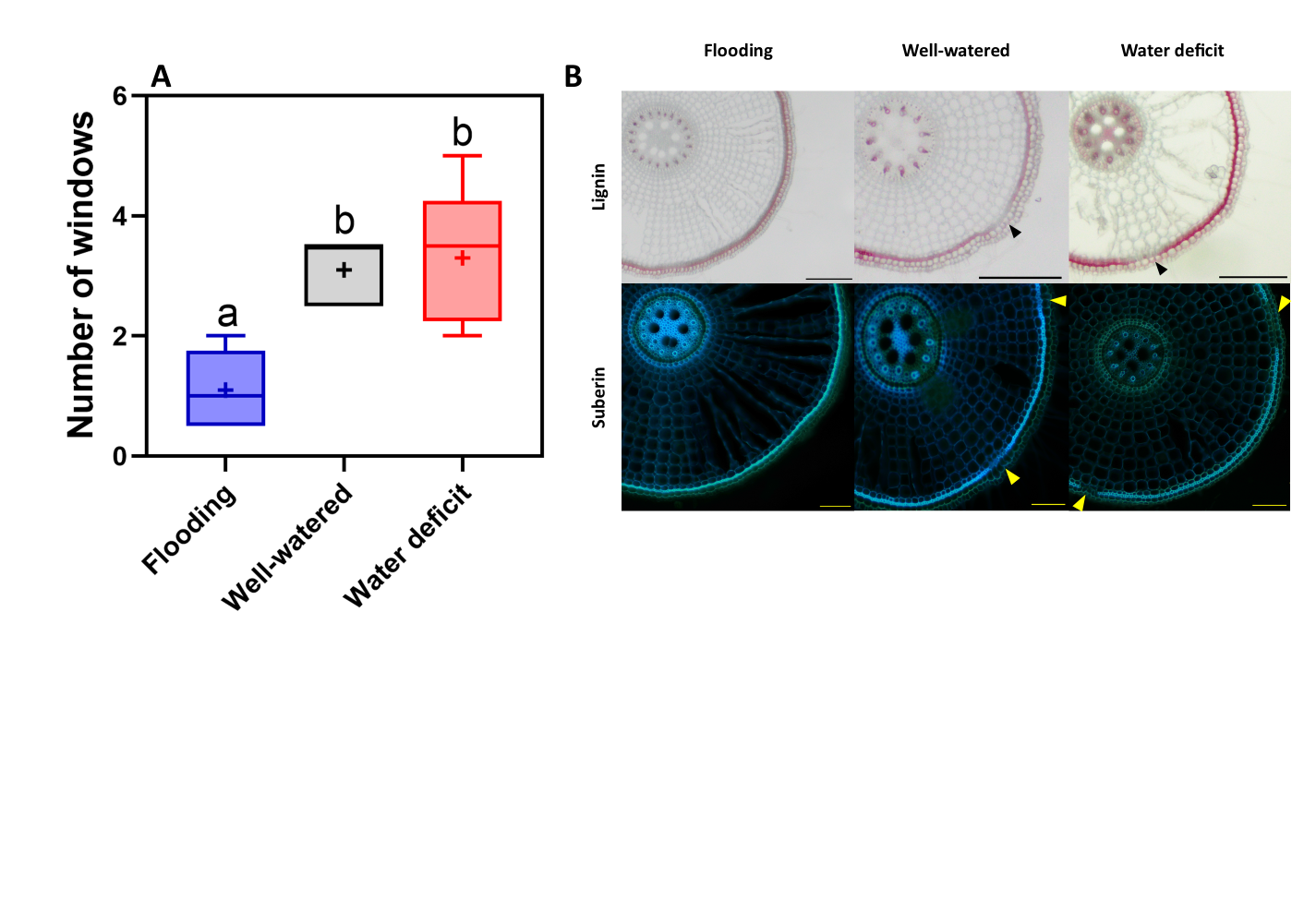
**Figure S4**

Number of windows without lignified or suberized cells (A). Representative sites for windows formation on roots of *O. longistaminata* grown in flooding, well-watered or water deficit conditions (B). The black arrows point at the window sites without lignification, whereas the yellow arrows point at the window sites without suberization. The windows were counted by identifying at least three consecutive cells within the cross-sections that lacked lignin or suberin deposits. The statistical comparisons were conducted with one-way ANOVA followed by Tukey’s test (different letters indicate significant difference, *P* < 0.05) and all data passed Shapiro-Wilk’s normality test. *n*= 5 for all. Mean, +; median, horizontal line; 2^nd^ and 3^rd^ quartiles, box; minimum and maximum values, whisker. Scale bar = 200 µm for lignin and 100 µm for suberin images.

## **Table S1**

Results of repeated measures ANOVA of responses of photosynthetic parameters to cutting of the rhizome connecting mother plant and ramet in *O. longistaminata. *, P ≤ 0.05; **, P ≤ 0.01; ***, P ≤ 0.001; ****, P ≤ 0.0001; ns, not significant.*

| Growth condition | Photosynthetic parameters | Source of Variation | % of total variation | P value summary |
| --- | --- | --- | --- | --- |
| Ramet under water deficit | Photosynthetic rate | Cutting | 24.3 | * |
|  |  | Plant | 29.9 | **** |
|  |  | Plant x cutting | 34.7 | **** |
|  | Transpiration rate | Cutting | 34.8 | ns |
|  |  | Plant | 11.9 | ns |
|  |  | Plant x cutting | 8.7 | ns |
|  | Stomatal conductance | Cutting | 47.2 | * |
|  |  | Plant | 5.8 | ns |
|  |  | Plant x cutting | 8.1 | ns |
| Mother plant under water deficit | Photosynthetic rate | Cutting | 33.4 | *** |
|  |  | Plant | 28.1 | * |
|  |  | Plant x cutting | 21.8 | ** |
|  | Transpiration rate | Cutting | 27.6 | * |
|  |  | Plant | 15.7 | ns |
|  |  | Plant x cutting | 13.1 | ns |
|  | Stomatal conductance | Cutting | 35.3 | *** |
|  |  | Plant | 14.4 | ns |
|  |  | Plant x cutting | 10.2 | ns |

## **Table S2**

Results of two-way ANOVA of root anatomical analyses in *O. longistaminata. *, P ≤ 0.05; **, P ≤ 0.01; ****, P ≤ 0.0001; ns, not significant.*

|  | Source of Variation | % of total variation | P value summary |
| --- | --- | --- | --- |
| Root diameter | Treatment | 72.1 | **** |
|  | Position | 5.7 | ** |
|  | Treatment × position | 0.6 | ns |
| Cortex to stele ratio | Treatment | 66.1 | **** |
|  | Position | 5.7 | * |
|  | Treatment × position | 1.2 | ns |
| Root porosity | Treatment | 19.0 | * |
|  | Position | 35.9 | **** |
|  | Treatment × position | 6.4 | * |
| Number of cell files | Treatment | 63.4 | ** |
|  | Position | 1.3 | * |
|  | Treatment × position | 0.4 | ns |

**Table S3**

Results of two-way ANOVA of 1^st^, 2^nd^, and 3^rd^ rhizome internodes from anatomical analyses of *O. longistaminata. *, P ≤ 0.05; **, P ≤ 0.01; ***, P ≤ 0.001; ns, not significant.*

|  | Source of Variation | % of total variation | P value summary |
| --- | --- | --- | --- |
| Rhizome diameter | Treatment | 68.4 | ** |
|  | Position | 13.3 | *** |
|  | Treatment × position | 0.7 | ns |
| Large gas spaces | Treatment | 65.1 | ** |
|  | Position | 17.5 | *** |
|  | Treatment × position | 2.0 | ns |
| Number of vascular bundles | Treatment | 53.6 | * |
|  | Position | 1.3 | ns |
|  | Treatment × position | 6.5 | ns |

## **Table S4**

Results of two-way ANOVA of 1^st^, 2^nd^, and 3^rd^ rhizome internodes anatomical analyses in *O. longistaminata. *, P ≤ 0.05; **, P ≤ 0.01; ***, P ≤ 0.001; ns, not significant.*

|  | Source of Variation | % of total variation | P value summary |
| --- | --- | --- | --- |
| Diameter of 1^st^ rhizome internode | Treatment | 73.5 | * |
|  | Position | 0.4 | ns |
|  | Position x treatment | 1.2 | ns |
| Large gas spaces of 1^st^ rhizome internode | Treatment | 56.0 | ns |
|  | Position | 0.9 | ns |
|  | Position x treatment | 0.7 | ns |
| Number of vascular bundles of 1^st^ rhizome internode | Treatment | 41.9 | ns |
|  | Position | 0.5 | ns |
|  | Position x treatment | 2.1 | ns |
| Diameter of 2^nd^ rhizome internode | Treatment | 85.0 | ** |
|  | Position | 0.1 | ns |
|  | Position x treatment | 0.0 | ns |
| Large gas spaces of 2^nd^ rhizome internode | Treatment | 89.6 | ** |
|  | Position | 0.1 | * |
|  | Position x treatment | 0.1 | ns |
| Number of vascular bundles of 2^nd^ rhizome internode | Treatment | 33.5 | ns |
|  | Position | 0.6 | ns |
|  | Position x treatment | 0.5 | ns |
| Diameter of 3^rd^ rhizome internode | Treatment | 84.4 | ** |
|  | Position | 0.4 | * |
|  | Position x treatment | 0.1 | ns |
| Large gas spaces of 3^rd^ rhizome internode | Treatment | 91.8 | *** |
|  | Position | 0.1 | ns |
|  | Position x treatment | 0.4 | ns |
| Number of vascular bundles of 3^rd^ rhizome internode | Treatment | 27.4 | ns |
|  | Position | 0.3 | ns |
|  | Position x treatment | 1.6 | ns |

## **Table S5**

Results of two-way ANOVA of radial water loss from roots and rhizomes of *O. longistaminata. ****, P ≤ 0.0001; ns, not significant.*

| Source of Variation | % of total variation | P value summary |
| --- | --- | --- |
| Treatment | 2.3 | ns |
| Tissue | 49.6 | **** |
| Tissue x Treatment | 29.9 | **** |

## **Table S6**

Results of two-way ANOVA of apparent permeance to O_2_ in roots and rhizomes of *O. longistaminata. *, P ≤ 0.05; **, P ≤ 0.01; ns, not significant.*

| Source of Variation | % of total variation | P value summary |
| --- | --- | --- |
| Treatment | 12.2 | * |
| Tissue | 23.6 | ** |
| Tissue x Treatment | 24.4 | ** |
